# Fecal MicroRNAs as Potential Biomarkers for Screening and Diagnosis of Intestinal Diseases

**DOI:** 10.3389/fmolb.2020.00181

**Published:** 2020-08-07

**Authors:** Humaira Rashid, Biplob Hossain, Towfida Siddiqua, Mamun Kabir, Zannatun Noor, Mamun Ahmed, Rashidul Haque

**Affiliations:** ^1^Emerging Infections and Parasitology Laboratory, International Centre for Diarrhoeal Disease Research, Dhaka, Bangladesh; ^2^Nutrition and Clinical Services Division (NCSD), International Centre for Diarrhoeal Disease Research, Dhaka, Bangladesh; ^3^Department of Biochemistry and Molecular Biology, University of Dhaka, Dhaka, Bangladesh

**Keywords:** fecal miRNA, intestinal barrier dysfunction, inflammatory bowel disease, colorectal cancer, qPCR, microarray, dPCR

## Abstract

MicroRNAs (miRNAs) are a class of conserved endogenous, small non-coding RNA molecules with a length of 18–25 nucleotides that regulate gene expression by RNA interference processes, including mRNA chopping, mRNA deadenylation, and translation inhibition. miRNAs maintain the physiological functions of the intestine and are instrumental in gut pathogenesis. miRNAs play an important role in intercellular communication and are present in all body fluids, including stools with different composition and concentrations. However, under diseased conditions, miRNAs are aberrantly expressed and act as negative regulators of gene expression. The stable and differentially expressed miRNAs in stool enables miRNAs to be used as potential biomarkers for screening of various intestinal diseases. In this review, we summarize the expressed miRNA profile in stool and highlight miRNAs as biomarkers with potential clinical and diagnostic applications, and we aim to address the prospects for recent advanced techniques for screening miRNA in diagnosis and prognosis of intestinal disorders.

## Introduction

**FIGURE 1:**
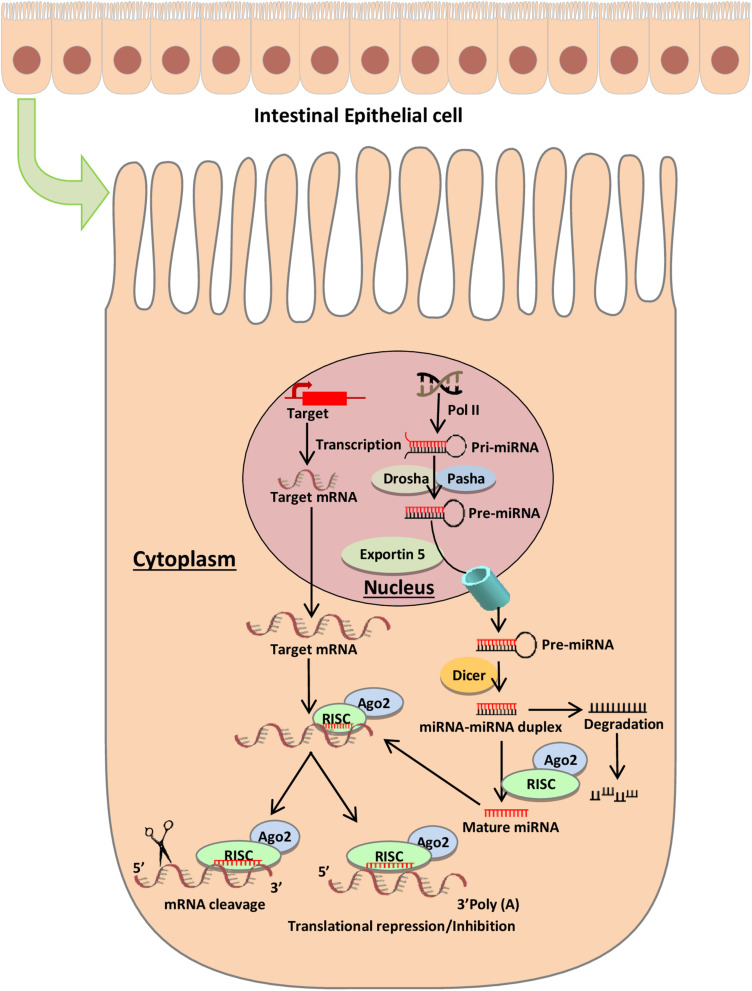
miRNA biogenesis and mode of action. The biogenesis of microRNAs begins in the nucleus where RNA polymerase II transcribes miR genes, generating a primary miRNA (pri-miRNA), which is subsequently cleaved by Drosha (RNase III endonuclease), resulting in a precursor miRNA (pre-miRNA) (Wan et al., 2016). The pre-miRNA is cleaved by the cytoplasmic endonuclease Dicer forming a duplex miRNA complex (O’Brien et al., 2018). The mature miRNA interacts with mRNA and induces the nuclease activity of RISC, thereby triggering translational repression or mRNA degradation.

MicroRNAs (miRNAs) are small non-coding RNAs in the range 18–25 nucleotides in length that act as regulators for post-transcriptional gene expression. The biogenesis of miRNAs is a complex multi-step process that begins in the nucleus (Figure 1). To form mature miRNAs, RNA polymerase II transcribes miRNA genes, to generate a primary miRNA (pri-miRNA), which is subsequently cleaved by Drosha (RNase III endonuclease), resulting in a precursor miRNA (pre-miRNA) (Wan et al., 2016). The pre-miRNA is cleaved by the cytoplasmic endonuclease Dicer and forms a mature miRNA duplex (O’Brien et al., 2018). One strand of the mature miRNA is loaded onto an Argonaute (Ago) protein forming a RNA-induced silencing complex (RISC) (Hammond et al., 2000; Wu et al., 2006). The mature miRNA functions by guiding the Ago protein and associated factors to target sites in the 3’ untranslated region (UTR) of mRNA. Typically, miRNA base-pairs with target mRNA, and the degree of complementarity between miRNA and target mRNA determines the repression of translation or reduction of target mRNA stability. A complete base pairing between the seed region of active miRNA and 3′ UTR of target mRNA promotes the degradation of mRNA, while the incomplete base pairing represses the translation initiation or elongation processes and promotes deadenylation followed by degradation of target mRNA in processing bodies (Valencia-Sanchez et al., 2006; Wu et al., 2006). miRNAs regulate the expression levels of thousands of target genes involved in different pathways and plays an important role in a variety of cellular and developmental processes (Ivey and Srivastava, 2015). miRNAs participate in intercellular communication and are isolated from body fluids, including stools (Moloney et al., 2018).

Although the function of extracellular miRNA in body fluids is not well characterized, the differentially expressed stable miRNAs in stool under diseased condition could be used as a potential biomarker for screening of intestinal diseases. In this review, we discuss the potential use of fecal miRNAs as molecular markers for screening and diagnosis of intestinal pathologies. Furthermore, we aim to address the prospects for recent advanced techniques for screening miRNA for specific disease in diagnosis and prognosis.

## Fecal miRNAs as Potential Biomarkers for Intestinal Barrier Dysfunction

Intestinal epithelial barrier is a complex anatomical and functional structure of gut lumen that allows the selective absorption of essential nutrients and fluids and acts as a barrier against toxins and microorganism invasions (Groschwitz and Hogan, 2009). Disruption of this barrier can trigger various intestinal diseases, characterized by increase in intestinal permeability (Fukui, 2016). It has been found that intestinal epithelial barrier dysfunction is more prevalent among the residents of low- and middle-income countries (LMICs) where the sanitation is often poor and hygiene practice is suboptimal (Mondal et al., 2012; Petri et al., 2014). Studies have revealed that children with enteric dysfunction have an impaired intestinal permeability that can lead to inflammatory bowel syndrome (IBS) and other inflammatory-related gut pathologies (Shulman et al., 2008). It is critically important to prevent the invasion of pathogenic microorganisms in order to maintain the integrity of gut epithelia. Repeated exposure to fecal pathogens, toxins, irritants, and pro-inflammatory cytokines can disrupt the tight junction’s barrier function, causing inflammation and facilitating the permeation of pro-inflammatory molecules such as unwanted colonial bacteria, bacterial antigens, and toxins to cross through and exacerbate the immune response (Fukui, 2016; Hossain et al., 2016). In different studies, it has been shown that infections caused by enteric protozoa, rotavirus, astrovirus, enterovirus, adenovirus, and enterotoxigenic *Escherichia coli* have an association with the aberrant expression of host cellular miRNAs that has a profound impact on child growth and development (Buret et al., 2002; Skalsky and Cullen, 2010; Zheng et al., 2013; Maudet et al., 2014; Ho et al., 2016; Zhou et al., 2016). miRNAs are highly relevant for infection-induced intestinal disorders as recent research has revealed their role in regulating the inflammatory responses and subsequently gastrointestinal diseases (Sheedy and O’neill, 2008; Bi et al., 2009).

Current evidence suggests that fecal miRNA could mediate dysbiosis-related inflammation of the host (Viennois et al., 2019). A previous study showed the perfect balance between microbiota composition and host metabolism and immunity (Liu et al., 2016). Alteration of gut microbiota composition is associated with intestinal barrier dysfunction that can cause inflammatory bowel disease (IBD) and colorectal cancer (CRC) (Belkaid and Naik, 2013; An et al., 2014; Yu, 2018). In addition, miRNAs have been shown to play a critical role in modulating viral infections such as rotavirus, enterovirus, which is largely responsible for gastroenteritis and alterations in enterocytes and bacterial microflora (Buret et al., 2002; De Magistris et al., 2003; Mondal et al., 2012; Maudet et al., 2014; Zhou et al., 2016).

**FIGURE 2:**
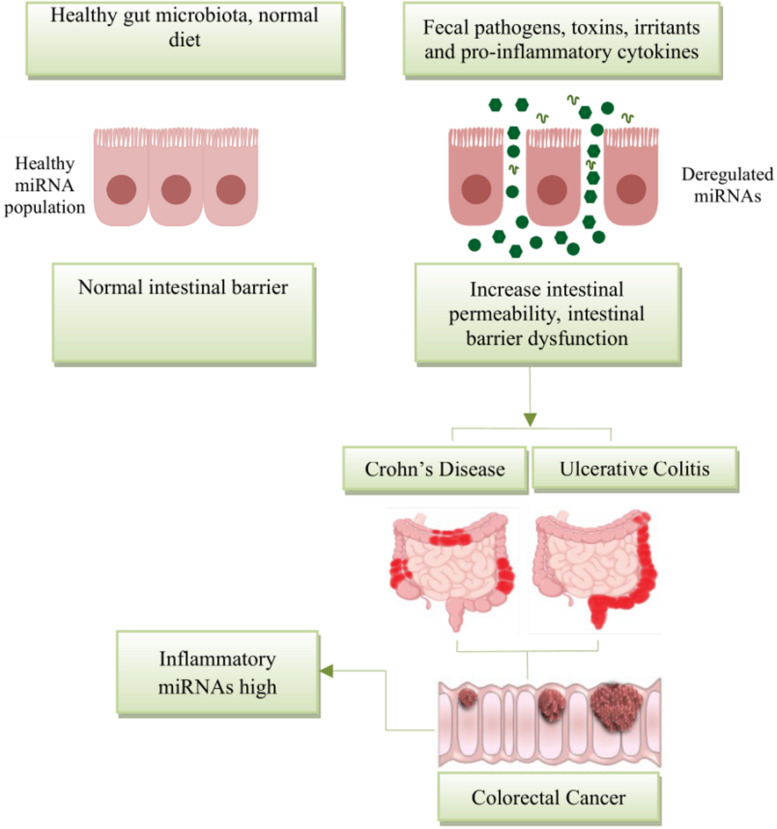
Schematic diagram represents the potential role of miRNA expression in intestinal diseases. The miRNA network plays a critical role in regulating gene expression in health and disease. miRNAs are essential regulators of inflammation. Post intestinal barrier damage, persistent inflammatory conditions can change the expression of microRNA, initiating or developing IBD. When IBD persists for a long period of time, the chronic dysregulation of miRNAs targets transcripts that encode intestinal barrier components and tumor-suppressor miRNAs, and this can lead in the disease development from IBD to CRC.

More recently, miRNAs have attracted a great deal of attention due to their role as key regulators of inflammation. Aberrant expression of miRNAs can play a pathogenic role in diseases, including those primarily affecting the gut (Figure 2). Cichon et al. (2014) reported that the intestinal barrier function is impaired in a typical Dicer1-deficient mouse model (i.e., absence of mmu-miR-192), resulting in spontaneous intestinal inflammation. Of note, miRNAs, such as miR-122a and miR-21, target negative regulators of immune response to promote inflammation (Ye et al., 2011; Zhang et al., 2015, 2017). Several investigations reveal that inflammatory cytokines such as tumor necrosis factor (TNF)-α and interferon gamma (IFNγ) may increase greatly the expression of miRNAs in enterocytes, cultured cells, and intestinal tissues (Ye et al., 2011; Haines et al., 2016). Lin et al. reported that TNF-α increased the expression of miR-21, which significantly increased barrier permeability (Zhang et al., 2015). Activation of these proinflammatory cytokines may induce barrier disruption and increased intestinal permeability.

The pathophysiological mechanisms of intestinal barrier dysfunction are multifaceted and not fully understood. Although miRNA has been mostly studied *in vitro* using cell lines, over the past few years, there is a growing interest in how miRNAs may act as a molecular marker to detect intestinal barrier dysfunction. Importantly, stool miRNA due to its stability, preservation, and high abundance may serve as potential biomarkers for screening and diagnosis of various intestinal disorders in patients in developing countries.

## Fecal miRNAs as Potential Biomarkers for Various Intestinal Diseases

Deregulation of miRNAs is linked with numerous diseases such as cancer (Lin and Gregory, 2015), hepatitis, cardiovascular (McManus and Ambros, 2011), neurodegenerative, and gastrointestinal diseases (Absalon et al., 2013). Studies have revealed that miRNAs can be secreted in body fluids including serum, urine, breast milk, and saliva, and in stool in encapsulated form within exosome (Turchinovich and Cho, 2014). Exosomes incorporating miRNA act as signaling molecules for mediating cell-to-cell communication and protects the miRNAs from degradation (Montecalvo et al., 2012; Wahlgren et al., 2012). miRNAs embedded in exosomes provide stability to miRNAs in feces and other fluids and protects from RNase degradation (Koga et al., 2011). The sensitive and specific detection of miRNAs in the extracellular environment has contributed to screening and diagnosis of therapeutic and prognostic biomarkers for multiple diseases. Numerous studies have revealed the association between dysregulation of miRNA and disease progression and validated miRNAs as potential biomarkers of neoplastic and non-neoplastic diseases.

Biomarkers are important for early detection of the disease and early detection could prevent malignancies and improve prognosis. Therefore, it is necessary and important to develop a method that would be affordable and safe with relatively high sensitivity and specificity. Approaches for the screening of IBD and CRC have been performed on feces like colonoscopies, flexible sigmoidoscopy, fecal occult blood test (FOBT), and the fecal immunochemical test (FIT). While colonoscopies and sigmoidoscopy are highly reliable screening tools for detecting IBD and CRC (Kato et al., 2016; Spiceland and Lodhia, 2018), they are invasive and costly, and thus impeding screening compliance. FOBT, which commonly detects blood in stool, is non-invasive and a better approach to screen IBD and CRC (Simadibrata, 2010); however, FIT, which measures the concentration of hemoglobin in feces by using an antibody that targets human hemoglobin, is recommended over FOBT as a non-invasive approach. These methods either have a significant sensitivity or specificity but not both (Link et al., 2010; Iannone et al., 2016).

MicroRNAs, which are remarkably stable and reproducibly detected even after a long-term storage of fecal samples for a number of years (Wohnhaas et al., 2020), are disease specific. Aberrant expression of fecal miRNAs in IBD and CRC explores miRNAs as potential non-invasive clinical biomarkers. Previous studies have isolated RNA from human stool and identified miRNAs as potential biomarkers for colon and pancreatic malignancies (Ahmed et al., 2009; Link et al., 2012). In another study, the total RNA isolation from stool and miRNA expression analysis has revealed that miR-21 and miR-106a are highly expressed in patients with CRC, indicating the feasibility of potential biomarkers for the development of non-invasive screening test of colorectal neoplasia (Link et al., 2010). In addition to diagnosis of cancer including distinguishing tissue origin or site, cancer subtypes, and detecting cancer at early stage, miRNA signatures can also facilitate to predict cancer prognosis, which has recently been suggested by a growing body of research (Li et al., 2010; Zhang et al., 2013). For example, the expression of fecal MiR-21 may be a potential and minimally invasive prognostic biomarker of CRC (Hibner et al., 2018), which need to be evaluated and validated in well-defined large cohorts of carefully selected cases.

Dysregulation of miRNA expression in intestine is related to different disease and provides the information to define new miRNA biomarkers; for example, the altered expression profile of fecal miRNA composition in active IBD patients versus control has been identified and fecal miR-223 and miR-1246 are found upregulated in the stool of active IBD patients (Verdier et al., 2019). Another study identified the highest expression of miR-21, miR-155, and miR-223 in feces of IBD patients and overexpression of miR-21 has been shown to cause intestinal epithelial barrier impairment. ROC curve analysis using miR-223 in feces showed that the diagnostic accuracy for active UC was (AUC: 0.93) with a specificity and sensitivity of 86.7 and 90%, respectively (Yang et al., 2013; Schönauen et al., 2018). Bastaminejad et al. (2017) showed that miRNA-21 expression in stool could be a promising biomarker for the diagnosis of CRC with a sensitivity and a specificity of 86.05 and 81.08% (AUC: 0.829), respectively.

Ahmed et al. (2009) initially detected the miRNA expression in the stool of CRC patients and identified the increased expression of seven miRNAs including miR-21, miR-106a, miR-96, miR-203, miR-20a, miR-326, and miR-92 and decrease in the expression of seven other miRNAs, miR-320, miR-126, miR-484-5p, miR-143, miR145, miR-16, and miR-125b, in the stool of CRC patients (Table 1). Ahmad et al. also selected a panel of fecal miRNAs where 12 miRNAs (miR-7, miR-17, miR-20a, miR-21, miR-92a, miR-96, miR-106a, miR-134, miR-183, miR-196a, miR-199a-3p, and miR214) were upregulated in the feces of CRC patients and 8 miRNAs (miR-9, miR-29b, miR-127-5p, miR-138, miR-143, miR-146a, miR-222, and miR-938) were downregulated in the feces of CRC patients. These miRNAs could differentiate CRC incidences from healthy controls and also different TNM stages with a high sensitivity and specificity (Ahmed et al., 2013). A comparative study of fecal miRNA expression profile by microRNA microarray demonstrated that the two miRNAs (miR-221 and miR-18a) that are highly expressed in stool of CRC patients with a sensitivity and specificity of 66 and 75%, respectively (AUC: 0.75) have a potential to be used as a non-invasive biomarker for screening of CRC (Yau et al., 2014). In addition, the significant decrease in the expression levels of miRNA-29, miRNA-223, and miRNA-224 have been detected in CRC patients, while in rectum cancer patients, the miRNA-29 is overexpressed (Zhu et al., 2016). The ROC curve analysis found that the AUC values were 0.777 for miR-29a with a sensitivity of 85% and a specificity of 61%, 0.649 for miR-223 with a sensitivity of 60% and a specificity of 71%, and 0.744 for miR-224 with a sensitivity and specificity of 75 and 63%, respectively.

**TABLE 1:** Key findings of studies involving fecal miRNA expression in patients with intestinal diseases.

Study type	Study participants	Age	Key findings	References
Proof-of-principle study using 2 independent patient cohorts	Patients: (i) CD: *n* = 39 (ii) UC: *n* = 18 (iii) Control: *n* = 15	Patients: 12–50 years (i) CD: 36.6 ± 12.3 years (ii) UC:44.9 ± 15.8 years Control: 53.5 ± 16.8 years	Higher expression in IBD stool samples was shown in miR-223, miR-155, miR-16, and miR-21.	Schönauen et al., 2018
Case–control study	Patients: (i) IBD: *n* = 10 (ii) Cancer: *n* = 15 (iii) Normal: *n* = 5	Adults	miR-21, miR-106a, miR-96, miR-203, miR-20a, miR-326, and miR-92 showed increased expression in stool of CRC patients and miR-320, miR-126, miR-484-5p, miR-143, miR-145, miR-16, and miR-125b showed decreased expression in stool (and also in tissue) of CRC patients. miRNAs from stool of UC patients was upregulated for four miRNAs (miR-21, miR-203, miR-126, and miR16), and downregulated for two miRNAs (miR-320 and miR-192).	Ahmed et al., 2009
Case–control study	Patients: (i) CD:(active: *n* = 22; inactive: *n* = 22) (ii) UC:(inactive: *n* = 11; active: *n* = 24) (iii) Control: *n* = 23	Patients: 27–69 years Control: 25–73 years	miR-223 and miR-1246 are present at high levels in stool and are increased in active IBD patients.	Verdier et al., 2019
Case–control study	Patients: *n* = 17 Healthy subjects: *n* = 28	Patients:46-80 years Healthy participants:36-79 years	Fecal miR-135b, miR-223 and miR-451 was significantly upregulated in CRC patients.	Phua et al., 2014
Case-control study	Patients: Adenoma: *n* = 151, Advanced Adenoma: *n* = 48, CRC: *n* = 198, Healthy subjects: *n* = 198	Patients: Adenoma: 60.39 ± 5.65 years Advanced Adenoma: 58.73 ± 6.78 years, CRC: 66.53 ± 11.05 years Healthy participants: 58.65 ± 6.87 years	Stool miR-221 and miR-18a were significantly upregulated in CRC stages I + II. There was no significant upregulation in adenoma or advanced adenoma for both miR-221 and miR-18a.	Yau et al., 2014
Retrospective case–control study	Patients: *n* = 80, Healthy subjects: *n* = 51	Patients: 60.7 ± 11.8 years Healthy participants: 49.3 ± 12.7 years	Fecal miRNA-29a, miR-223, and miR-224 was significantly down regulated in the stools of the CRC patients.	Zhu et al., 2016
Case–control study	Patients: (i) Adenoma: *n* = 110 (ii) Advanced adenoma: *n* = 59 (iii) CRC: *n* = 104 (iv) IBD: *n* = 42 (v) Healthy participants: *n* = 109	Patients: (i) Adenoma:58.9 ± 6.9 years (ii) Advanced adenoma: 62.1 ± 9.5 years (iii) CRC: 66.8 ± 11.9 years (iv) IBD: 48.2 ± 11.6 years (v) Healthy participants: 60.4 ± 7.0 years	Significantly higher level of miR-135b, but not miR-31, was detected in the stool samples of CRC (*P* < 0.0001) and advanced adenoma. Relatively low level of miR-135b detected in stool of patients with IBD.	Wu et al., 2014

In particular, miRNAs, being a robust biomarker for CRC and IBDs, provide an opportunity to be used as a non-invasive biomarker to detect various intestinal diseases with a high sensitivity and specificity.

## Experimental Approaches for Isolation, Detection and Quantification of Fecal miRNA

MicroRNAs have been detected in almost all body fluids including stool (Weber et al., 2010; Moloney et al., 2018), in a remarkably stable form. The stability of miRNAs plays an important role in shaping miRNA expression profile and makes it feasible for accurate measurement and detection. Several techniques have been developed to analyze the altered miRNA expression profile and the identification of targets in different intestinal diseases. The specific and sensitive methods for extraction and detection reveal the potential of miRNAs in diagnostics, prognosis, and therapy.

### miRNA Isolation From Fecal Samples

There are several methods used in different studies for miRNA extraction from biofluids including stool samples (Iborra et al., 2013; Wu et al., 2014; Ren et al., 2015). To provide high-quality miRNA, a number of studies have focused on the technical optimization of miRNA extraction. Guanidinium-phenol-based solutions, such as TRIzol/TRI reagent, are a convenient and effective method for miRNA extraction from stool samples. The TRIzol method is cost-effective, which yields good-quality miRNA, removes the inhibitors, and does not rely on specialized equipment (Paula et al., 2003). The application of the TRIzol/TRI-Reagent after bead beating followed by alcohol precipitation is the most widespread method for the isolation of total RNAs including miRNAs. However, miRNA extractions using the TRIzol method have the risks of cross-contamination of phenol, DNA, lipid or proteins. Combination of TRIzol-based lysis and spin column-based extraction has been found to yield high, ultra-pure miRNA for sensitive downstream processes (Yau et al., 2014). miRNeasy extraction kit in combination with phenol/guanidine-based lysis has strong efficiency in removing inhibitors, exogenous and intrinsic, and the purification of RNA greater than 18 nucleotides for downstream applications. Other commercially available kits – Norgen’s Stool Total RNA Purification Kit, Macherey-Nagel’s NucleoSpin RNAs stool kit, E.Z.N.A^TM^ stool RNA Kit (OMEGA, GA, United States), etc. – have been used in different studies for the extraction of ultra-pure high-quality miRNAs (Ren et al., 2015). For exosomal RNA extraction, the immunomagnetic beads conjugated with target antibody are used for exosome isolation. The exosomes isolated are homogenized and miRNAs are extracted using the RNA extraction kits for downstream applications (Koga et al., 2011). Additional optimization and validation of extraction procedures is encouraged for high extraction efficiency and purity of miRNAs (Tarallo et al., 2014). miRNA extracted from the patient stool can then be used for quantification and miRNA profile analysis.

### Detection and Quantification of Fecal miRNAs

#### Quantitative Real-Time PCR (qPCR)

The screening and diagnosis of certain diseases rely heavily on invasive endoscopic techniques, which have several practical limitations such as bowel preparation and sedation. Therefore, an optimal screening procedure, preferably non-invasive diagnostic technologies, has a tremendous potential to increase survivorship. Many studies have reported and investigated the potential of miRNAs as diagnostic biomarkers where an efficient, robust, and standardized method for miRNA detection has been successfully produced to profile miRNAs.

Quantitative real-time polymerase chain reaction (qPCR) is a commonly used method for quantifying miRNA expression. Due to the small size, the traditional qPCR cannot be applied for quantification of mature miRNAs. Therefore, it is important to extend the length of mature miRNA before performing the qPCR for amplification and quantification. The stem-loop reverse transcription PCR is the most common method used for quantification of miRNA. This method uses stem-loop primer specific for the synthesis of first-strand cDNA of mature miRNA. The cDNA synthesized can be amplified using miRNA-specific forward primer and stem-loop reverse primer. The sensitive, specific miRNA hydrolysis TaqMan probe is used for fluorescence and quantification of mature miRNA expression (Chen et al., 2005). In another method, poly(A) polymerase is used to polyadenylate the 3′ end of all mature miRNAs, and the oligodT primer that has an adapter sequence at the 5′ end is employed to generate a cDNA (Shi and Chiang, 2005). A forward primer specific to miRNA and a universal reverse primer complimentary to adapter sequence is used for PCR amplification and SYBR Green-based real-time PCR for quantification (Ro et al., 2006). However, the traditional mature miRNA-qPCR has problematic issues when trying to discriminate closely related miRNAs that differ only in a few of the bases or when the RNA yield is very low. The Locked Nucleic Acid (LNA) primers have been developed that are remarkably specific to targets compared to DNA and RNA probes and can discriminate single-nucleotide mismatch (Owczarzy et al., 2011). A unique combination of two microRNA-specific LNA primers further increases specificity toward the detection of diagnostic biomarkers in challenging samples like stool. SYBR Green- and TaqMan qPCR-based expression analyses of stool miRNA were able to monitor changes at various stages of CRC, IBDs, and other intestinal diseases, allowing for reliable diagnostic screening of diseases (Ahmed et al., 2009, 2018b; Wu et al., 2012; Schönauen et al., 2018). The miRNA qPCR is a reliable screening method for biosignatures in clinical specimens with a high sensitivity for detection.

#### Microarray Profiling

Microarray-based expression analysis is a well-established, powerful, high-throughput, cost-effective technique capable of identifying a large number of candidate miRNA expression levels in one assay within a large number of samples processed in parallel in a single experiment (Thomson et al., 2007). Microarray has been extensively used to measure the expression profile of miRNA in stool (Link et al., 2012; Phua et al., 2014). For routine expression profiling, microarray is one of the most recent well-established hybridization techniques that utilize DNA probes to detect specific miRNAs (Liu et al., 2004; Wang et al., 2007). In miRNA microarray, the miRNAs are isolated and labeled with fluorescent dye followed by hybridization with corresponding immobilized microRNA probes on the glass slide (Li and Ruan, 2009). The fluorescence signals emitted from labeled miRNA bound at different spots can be detected.

The use of microarray for clinical diagnostics has been under development for several years. Currently, a variety of microarray systems have been customized for miRNA quantification including GeneChip (Affymetrix), TaqMan^TM^Array (Applied Biosystems), miRCURYLNA (Exiqon), and SurePrint (Agilent) (Phua et al., 2014; Wu et al., 2014; Yau et al., 2014). These platforms offer unique advantages in the specific and sensitive detection of miRNA sequences. The probes are designed for specific mature miRNA sequences where the major differences in these platforms include hybridization, washing procedures, and fluorescent dyes. Although miRNA microarray (microchip) is a common method for evaluating known miRNAs, this technology also has some drawbacks. For example, it cannot be utilized for absolute quantification and has a comparative less sensitivity, and is usually considered to have a higher background signal and lower dynamic range than qPCR (Table 2; Reid et al., 2011; Zampetaki and Mayr, 2012). The number of miRNA species that can be detected in a reaction is small and therefore the method of normalization can influence the result (Moldovan et al., 2014).

**TABLE 2:** Advantages and limitations of techniques used to quantify fecal miRNA.

Method name	Advantages	Limitations	References
qPCR	– Gold standard for quantification of gene expression. – A large dynamic range real-time monitoring. – Accurate method for miRNA quantification.	– Controversy due to different results. – Genome coverage is very limited.	Heid et al., 1996; Chen et al., 2005; Schmittgen and Livak, 2008; Balcells et al., 2011; Yong et al., 2013; Ahmed et al., 2017
Microarray	– Inexpensive, robust, and reliable. – Ease of use, availability of platforms, and lower cost relative to other exploratory methods.	– Lack of rigorous standards for data collection, analysis, and validation. – Cannot be utilized for absolute quantification and have a comparative less sensitivity. – Is usually considered to have a higher background signal and lower dynamic range than qPCR.	Nakanishi et al., 2001; Conway and Schoolnik, 2003; Russo et al., 2003; Jaluria et al., 2007
Digital PCR	– Can tolerate PCR inhibitors. – Time- and reagent-efficient. – Sensitive and precise absolute quantification.	– More costly than other detection methods.	Brunetto et al., 2014; Rački et al., 2014; Zhao et al., 2015; Stein et al., 2017; Ahmed et al., 2018a; Quan et al., 2018

#### Digital PCR (dPCR) for Quantifying miRNA in Fecal Samples

Digital PCR is the latest technology in the PCR arsenal. It is a highly precise approach, and the driving innovation behind it is sample partitioning where each partition serves as an individual PCR reaction. Digital PCR can amplify a single molecule a million-fold. In practice, it is well suited to applications requiring accurate quantification, excellent reproducibility, and sensitivity. dPCR can be used as a validated miRNA diagnostic stool test to screen for intestinal-related diseases (Ahmed et al., 2019). This newly introduced approach provides an alternative technique to qPCR for absolute quantification of miRNA. The Quant Studio^TM^ 3D Digital PCR System and Quant Studio 12K Flex can read the digital chip that contains 20,000 reaction wells in less than 1 min, following thermal cycling. Bio-Rad’s droplet PCR is currently commercially available as a third-generation PCR technology. This method, claimed to be of an extraordinary sensitivity, utilizes nanodroplet sample partitioning (Miotto et al., 2014). Droplet dPCR does not require the calibration curves for sample quantification. The digital PCR has the reaction tolerance to PCR inhibitors and is more reproducible than qPCR (Hindson et al., 2013), which is an important advantage on using clinical samples like stool (Sedlak et al., 2014). dPCR assay uses a nanofluidic chip and represents an easy and simple method to run in parallel up to millions of PCR reactions. Ahmed FE et al. reported the analysis of absolute miRNA expression from stool samples by a chip-based dPCR test. The results showed that from a selection of 14 miRNAs, 12 of them showed increased while 2 showed decreased expression (Ahmed et al., 2019). Following the end point PCR, Poisson statistical analysis is applied to determine the absolute quantity of the model containing the numbers of positive and negative observed reactions. The digital PCR approach has several potential benefits over real-time PCR and microarray. This novel method has high sensitivity, precision, and the capability to obtain absolute quantification without external references, making dPCR superior over other methods for many important applications including copy number variation (CNV) and expression analysis.

Samples such as stool that show high inhibition can be assayed using a dPCR, thereby offering a high-throughput and affordable quantitation (Yang et al., 2014). Therefore, dPCR has the potential to have a considerable impact on research, diagnostic, prognostic, and predictive test applications.

## Challenges for Quantifying miRNA in Stool Samples

The evaluation of miRNA expression profile in stool samples has applications in diagnosis of several human diseases. However, there are challenges that need to be overcome in order to use miRNAs for this purpose. The following research activities are recommended in order to advance miRNA-guided diagnostics:

(i) Designing innovative quantification methods and also improving existing miRNA detection technologies to get better sensitivity and specificity of the test performed.
(ii) Evaluation of cross-platform comparisons of methodologies applied for miRNA detection in stool samples.
(iii) Designing and developing user-friendly online resources to analyze and compare miRNA expression data.
(iv) Conducting a feasibility study for a potential knowledge-based system application of miRNA testing in stool samples for regular use in clinical practice.

## Conclusion

Understanding various intestinal diseases requires thorough investigation about the alteration of miRNA functioning and disease progression. An association between alteration of miRNA expression profile and progression of intestinal diseases provides the opportunity to explore new biomarkers in stool samples. So far, biomarkers have been identified in stool for different diseases and new techniques are being developed for high-throughput screening of miRNAs in stool for the diagnosis and prognosis of disease. However, the data on the alteration of miRNA in intestinal diseases and differential profile of miRNA in stool is not complete; more investigation and screening are required for the exploration of new biomarkers.

## Author Contributions

HR conceived and designed the study and drafted the manuscript. HR, BH, RH, and MA reviewed the first draft of the manuscript and suggested additional analysis. HR, BH, TS, ZN, MK, MA, and RH revised the final draft manuscript and provided critical comments. All authors read and approved the final version of the manuscript.

## Disclaimer

The authors alone are responsible for the views expressed in this manuscript.

## Conflict of Interest

The authors declare that the research was conducted in the absence of any commercial or financial relationships that could be construed as a potential conflict of interest.
